# Potentials and challenges of next generation sequencing-guided individualized treatment for management of rifampicin-resistant tuberculosis – A qualitative study

**DOI:** 10.1371/journal.pone.0350514

**Published:** 2026-06-26

**Authors:** Tu Pham Hien Trang, Antonio Barrenechea-Pulache, Katinka De Wet, Boitumelo Fanampe, Salome Charalambous, Annelies Van Rie

**Affiliations:** 1 Department of Family Medicine and Population Health, Faculty of Medicine and Health Sciences, University of Antwerp, Antwerp, Belgium; 2 Facultad de Medicina, Universidad Científica del Sur, Lima, Perú; 3 Interdisciplinary Centre for Digital Futures/ Department of Sociology, University of the Free State, Free State, South Africa; 4 Free State Department of Health, Bloemfontein, South Africa; 5 Aurum Institute, Johannesburg, South Africa; St Petersburg Pasteur Institute, RUSSIAN FEDERATION

## Abstract

**Background:**

Next generation sequencing (NGS) is a powerful technology for diagnosing and managing rifampicin-resistant tuberculosis (RR-TB). To facilitate its integration into routine RR-TB care, a treatment recommendation system was developed to automatically translate whole genome sequencing (WGS) results into treatment recommendations. The effectiveness of WGS-guided treatment recommendations was evaluated in the SMARTT trial.

**Methods:**

We conducted a focus group discussion among eleven health care workers (HCWs) involved with managing SMARTT trial participants to explore their experiences and attitudes towards WGS-guided treatment recommendations. Thematic analysis was undertaken with an inductive approach.

**Results:**

The use of WGS and automated treatment recommendations simplified drug susceptibility testing (DST) and was perceived as beneficial to patients and HCWs. The strategy did not negatively impact HCWs autonomy and competence. Trust and acceptance were compromised by slow turn-around time, guideline deviations, discordance between DST methods, and unfavorable outcomes of some patients. Implementation challenges included drug stock-out, limited internet connection and training requirements.

**Conclusion:**

NGS accompanied by automated treatment recommendations holds promise for improving RR-TB care in high-burden settings, benefiting both patients and HCWs. Perspectives of policymakers, laboratory staff and patients should be explored to support the routine implementation of NGS in RR-TB management.

## Introduction

Tuberculosis resistant to rifampicin (RR-TB) is difficult to treat, with a treatment success rate of 63% compared with 88% for drug-sensitive TB [1]. The approval of bedaquiline (BDQ) in 2012 allowed the development of short all-oral regimens that were recently endorsed by the World Health Organization [2]. Unfortunately, BDQ resistance was reported soon after its introduction [3].

Currently, in the absence of rapid drug susceptibility tests (DST) for BDQ, a standardized treatment regimen is empirically started, and a rescue regimen is selected based on incomplete DST information when BDQ resistance is diagnosed [4,5]. Next-generation sequencing (NGS), including whole-genome sequencing (WGS) and targeted NGS (tNGS), can rapidly determine a comprehensive *Mycobacterium tuberculosis* (*Mtb*) drug resistance profile [6,7]; however, NGS is rarely applied in high RR-TB burden settings. Even when doctors have access to NGS, there is limited expertise in translating NGS results into individualized treatment regimens [8,9].

An automated treatment recommendation tool was developed using machine-learning methods to facilitate the effective use of NGS data for RR-TB management [10,11]. The effectiveness of WGS and automated treatment recommendations was evaluated in the pragmatic *Sequencing Mycobacteria and Algorithm-determined Resistant Tuberculosis Treatment* (SMARTT) trial [12]. We conducted a focus group discussion (FGD) among healthcare workers (HCW) involved in managing trial participants to assess their experiences and attitudes towards the use of WGS and automated treatment recommendations for the management of RR-TB.

## Methods

This qualitative study was included in the SMARTT trial, a pragmatic trial conducted in the Free State province of South Africa. The study setting of the trial was described in detail elsewhere [12,13]. Total population purposive sampling was employed, i.e., all physicians and nurses who managed the SMARTT trial participants were invited to participate. The FGD method was chosen because the interactive format allowed participants to build on each other’s responses and reflect on collective or diverging practices and perspectives, and trigger deeper reflection. A FGD was considered appropriate as the discussed topics were not sensitive and participants had similar professional roles, facilitating open discussion within a relatively homogeneous group. One single FGD was organized given to the small number of eligible participants able to participate. Written informed consents were obtained from all participants prior to participation in the study, all contributions were voluntarily provided, and the findings were recorded in a way to anonymize responses. The in-person FGD was conducted at time of a provincial meeting in November 16^th^ 2022. The FGD was led by an independent and experienced qualitative researcher (KDW). The FGD guide (provided in Supplementary material in S1 File) was informed by the technology acceptance model and the findings of studies on the uptake of computerized clinical decision support systems, artificial intelligence, and WGS [14–19]. Key discussion topics were the experience with the use of the treatment recommender for WGS-guided RR-TB regimen selection, perceived benefits, risk, facilitators and barriers of adopting WGS-guided treatment and the treatment recommender in clinical practice*.*

The FGD was recorded and transcribed verbatim. NVIVO Software (version 1.6.1) was used for the thematic analysis. An inductive approach was employed, with data familiarization, generation of initial open codes, categorization of codes into (sub)themes, identification of interactions between (sub)themes, and mapping of themes into an articulate story. Two researchers independently conducted the open coding (TPHT and AB), the codes were then compared and combined to reach consensus on the themes and subthemes. The results were discussed with the SMARTT study team in order to interpret the findings.

### Researcher reflexivity

KDW (PhD) is a medical sociologist based in the geographical setting where the trial was implemented. Understanding healthcare worker perspectives on novel RR-TB management approaches relates to her research focuses on the role of digital innovations in improving health and social outcomes. She was not acquainted with the participants prior to the FGD and was not otherwise involved in the SMARTT trial. TPHT (MSc) is an epidemiologist with formal training in qualitative research. She was involved in the implementation of the SMARTT trial, which might have influenced her prior favourable views regarding the potential utility of WGS. AB (MD, MSc) is an epidemiologist with formal training in qualitative research and a medical doctor with experience working in resource-constrained settings. He was not otherwise involved in the trial. To minimize personal or professional biases on data interpretation, coding and theme development were discussed both between the two researchers performing the coding and also with a broader SMARTT trial team to challenge assumptions and reach consensus on interpretation that more accurately reflects the context-specific experiences.

### Ethics approval

The study was approved by the ethics committee of the University Hospital Antwerp and the University of Antwerp (reference number 3694) and University of the Free State (UFS-HSD2019/0364/2004). The reporting of this study conforms to the Standards for Reporting Qualitative Research (SRQR) [20].

## Results

### Participants’ profile

Among the 11 FGD participants, six were physicians, four were nurses trained in the management of multidrug-resistant TB (MDR-TB), and one was an MDR-TB health district coordinator. The participants were between 29 and 64 years of age, and six were female. The duration of the FGD was one hour 32 minutes.

### Overview of themes

Five key themes emerged during the FGD. HCWs discussed the potential of NGS to improve drug susceptibility testing, the potential of NGS-guided treatment to improve treatment the care of people with RR-TB, the impact of NGS-guided treatment recommendations on the physician’s autonomy and competence, the trust in and acceptance of NGS-guided treatment recommendations, and the observed and expected challenges when implementing NGS-guided care into routine RR-TB management.

### Theme 1: Improved and faster Mtb drug susceptibility testing

According to the participants, the WGS simplifies the DST algorithm by reducing the number of tests and the time required for a comprehensive *Mtb* drug resistance profile.

*“The process of getting a DST […], it takes quite long. You have to go through emailing someone getting approval. So, with this –the whole genome sequencing – you pretty much know what drug works, and what does not work.”* (P#2)

*“This is a genetic study of the bacillus that can be done very rapidly. […] the LPA [line probe assays] will sometimes take six weeks, then we get the results. At this time, something wrong could go with the patient.”* (P#9)

One HCW suggested reserving WGS for complex cases, while most HCWs preferred WGS over line probe assays (LPA) for all RR-TB patients.

*“If there is resistance to fluoroquinolones (FQ), the patient is already XDR, then it is better to have the genome strategy than the LPA, and to get short-time result.”* (P#9)

*“I think the first one [performing WGS for all RR-TB patients] will be cost-effective because from the start we will be knowing what we are dealing with […] So, we are not going to waste any money because with LPA you will be adding cost upon cost.”* (P#6)

One HCW suggested that WGS would be beneficial for all types of TB.

“It is reserved to the pulmonary, but scientifically it can be used for extra-pulmonary […] it would give us very nice results and guide us to the drugs that we can use.” (P#9)

### Theme 2: Improved RR-TB patient care

Several participants stated that accompanying WGS results with treatment recommendations aid them in interpreting the *Mtb* strain’s genomic resistance profile, which in turn reduced the complexity of decision-making and allowed evidence-based informed decision.

*“It is able to assist the patient by showing doctor ‘this is what is supposed to be happening; this is the recommender […] we are doing this and this”.* (P#5)

*“What is recommended is evidence-based from the susceptibility of drugs and the resistant mutations that exclude certain drugs.”* (P#8)

*“It makes a difference because you know what you are dealing with […] because you will be having the whole resistance pattern of the patient.”* (P#2)

The approach was viewed as a novel and more dynamic treatment strategy.

*“This is a dynamic matter, compared to the old way of treating […] This is the way that we will go, we start the patient in the adequate treatment, and […] change because the patient – the DNA of the bacillus […] It is dynamic, and it moves to the best treatment for the patient.”* (P#9)

A shorter treatment duration and reduced pill burden were viewed as major benefits for patients and HCWs. The notable perceived advantages included improving patient’s adherence, facilitate adverse event management, and reducing HCWs’ workload associated with patient management.

*“It reduced the duration of how long a patient takes the medications, which obviously reduces our workload.”* (P#2)

*“The reduction of pill burden is beneficial both to the doctor and the patient, and, you know, it makes adherence much better.” (*P#11)

*“So, if the reduction, because of the whole genome sequencing, one is able to see which specific side effects will be from this treatment […] Unlike if it’s a whole seven core drugs, you are not sure which one is causing this. It is much better.”* (P#5)

### Theme 3: Impact on physician’s autonomy and competence

A commonly shared sentiment among the participating HCWs was that they still bore full responsibility and autonomy despite using the treatment recommendation tool. They critically evaluated the regimens and remained vigilant, particularly when the patient’s status deteriorated.

*“The machinery, it gives to human power and authority. […] but it will never replace the human brain.”* (P#9)

*“Is it really the machine, because machines are very perfect, or is it thinking? […] I was asking myself, can we trust really the recommended regimen? Or can the clinician also focus on what he’s thinking about the patient?”* (P#1)

*“There is nothing 100%. It can put us in the wrong way. That’s why we need to re-evaluate, and to see as a clinician how the evolution of the patient is.”* (P#9)

*“In case the patient is changing condition, there is something new […] it’s like we criticize ourselves ‘this thing, why is it not doing well’ […] Then we adapt.”* (P#1)

The HCWs viewed the treatment recommendation system as a way to complement guidelines for treatment individualization. Some however raised concerns that the convenience of a treatment recommendation could create a level of dependency, especially for less experienced physicians.

*“I think this won’t really affect your competence. […] We still have to prescribe according to the guidelines […]. So, I don’t really see a problem. All that it does is individualizing the patient care, it’s not like, you know, just giving everybody the same treatment.”* (P#10)

*“But to someone who might not be trained, they might be getting into the practice of just prescribing what the recommender says.”* (P#2)

### Theme 4: Trust in and acceptance of RR-TB treatment recommendations

Some participating HCWs remarked that due to limited knowledge on mutations, they felt that they had to rely on the conclusions of the report.

*“Personally, there are a lot of those resistance patterns that I do not know. So, I am just given this information, and I have to trust what it says.”* (P#2)

HCWs questioned the value of an individualized regimen when the patient responded well to the standard regimen. Additionally, they might doubt the strength of the recommended regimen when the recommended regimen was not considered “strong enough” which is to say when it does not include a sufficient number of Group A drugs.

“Sometimes, when the recommendation comes, the patient has already advanced in treatment.” (P#10)

“I trust it to a certain extent, especially when the recommended treatment has what we call ‘powerful’ drugs in it, or drugs that are classified higher on the hierarchy of MDR drugs. And I trust it less when the so-called drugs which are lower on the classification, more of them are put on the recommended treatment. Then that’s when one has to decide whether you go with the recommended, or do you rather go with the standard care of treatment?” (P#8)

HCWs were uncomfortable when the treatment recommendation deviated from the guidelines. For instance, physicians were hesitant to combine moxifloxacin with bedaquiline.

“We don’t use bedaquiline and moxifloxacin together. […] According to our guidelines, you use Levofloxacin instead.” (P#2)

The occurrence of relapse in patients receiving individualized treatment negatively impacted trust in the system.

“You have a patient relapsing - who completed the six months of the individualized regimen, […], obviously they are promised the four best drugs that work […] you start to query ‘was it the genome sequencing result?’” (P#2)

Discordant results between LPA and WGS also caused confusion.

“Like we find that the patient is rifampicin-resistant and then you’ll take those sputum samples for genome sequencing, and then the patient is not MDR […] we have started the patient on treatment, and it is very difficult for those people that refer the patient to you.” (P#6)

### Theme 5: Implementation challenges

Several participants mentioned that they had experienced that the drugs included in the recommended individualized regimen were not available because of stockouts, making it not possible to implement the recommended regimen

“Sometimes you find that the drugs… they’re just not there.” (P#10)

Some HCWs were not notified when treatment recommendation results were available or did not have the internet at work.

“There were a few bumps where we might not be notified in time that the result is available.” (P#2)

“Where we work there is no network. The results are there, and I must reply. So, you must go where there is a network to find the result.” (P#1)

One HCW recommended that a downloadable app would be more convenient than a link sent via email.

“I’m not tech-savvy, but in terms of accessing the app we constantly have to follow a link that we are sent to my emails. […] So maybe if the app could be…downloadable” (P#2)

Another stated challenge was frequent staff changes, which could disrupt patient management because using the treatment recommendation system requires training.

“if the person who is trained is not around, then it is a problem to get to the result” (P#1)

“So, maybe the same clinician might not be following up the patient, someone else could manage the patient thereafter, and it would be pertinent to know what happened.” (P#2)

## Discussion

The adoption of NGS in routine care could improve RR-TB management but also presents challenges due to limited understanding, low trust, poor sustainability, and technical barriers^21^. In our study, most HCWs viewed WGS with automated treatment recommendations as transformative by simplifying the DST, enabling evidence-based decisions, and creating potential benefits for both HCWs and patients. The novel NGS-guided approach was viewed as evidence-based and dynamic, enabling HCWs to switch from a standard regimen to an individualized treatment that reduces workload, pill count, and drug toxicity. Nevertheless, some hesitance remained due to limited understanding of NGS, particularly when the recommendations deviated significantly from the guidelines and did not include Group-A drugs. HCWs did not perceive that automated treatment recommendations threatened their autonomy or competence. The overall trust in the system was high, but was challenged in instances of DST discordance or when treatment recommendations deviated from the guidelines. Limited internet connections and training requirements were raised as implementation challenges.

Our findings contrast with those of a study from Madagascar and Canada, where HCWs indicated uncertainty about how WGS would complement existing technologies such as Xpert MTB/RIF, LPA, and phenotypic DST [21]. This difference likely reflects the hands-on WGS experience of our participants compared with the limited exposure of HCWs to WGS in those settings. Additionally, South Africa has a high RR-TB burden; therefore, healthcare workers are likely to experience more challenging cases. The participating HCWs felt empowered by the WGS-guided treatment recommendations. They overcame the limitations of WGS reported in a South African study, where HCWs required support to interpret mutations and manage the results [22].

The ethical implications of artificial intelligence (AI)-based decision support systems are of concern, especially regarding their impact on HCWs’ autonomy and competency [23,24]. In our study, HCWs did not feel that WGS-guided treatment recommendations affected their authority, as “the machinery gives power and authority… but it will never replace the human brain”. HCWs stated that they were ultimately the ones choosing to follow the recommendation or not. The treatment recommender tool thus achieved the intended impact as a decision aid, and HCWs did not feel overshadowed or replaced [25]. HCWs also indicated that they remained critical and vigilant, adapting regimens based on the patient’s response to treatment, confirming that trust in AI in healthcare is enhanced when accompanied by a healthy dose of skepticism and criticism [26]. Nevertheless, automated recommendations could reduce competence in less experienced HCWs if followed uncritically. These sentiments echo the need of adapting medical education in the face of tools that can cognitively surpass humans.

Performance expectancy is crucial for acceptance of digital intervention [16]. For *Mtb* NGS, a study found that “people get a little bit too focused on perfection and do not realize that every other test used in the past is nowhere near perfect” [21]. We found a similar sentiment, as trust was diminished when one patient did not respond well to the recommended treatment, or when the WGS and LPA results were discordant. Similar concerns regarding discordance have also been noted in other studies [22]. These high expectations highlight the importance of robust evidence, transparency, and a clear demonstration of the clinical impact of NGS [21,26].

Important limitations to the implementation of WGS and automated treatment recommendations were noted. First, the turn-around time of culture-based WGS results made it difficult for HCWs to alter the empirical regimen, especially when the patient was responding well to treatment. The approval of tNGS and the recent launch of a culture-free kit for WGS may address this issue [27]. Second, skepticism arose when the recommended regimen deviated from the guidelines. In response, we revised the treatment recommender to only individualize the regimen when resistance to one of the BPaLM drugs was detected. Third, as training was identified as a critical component of implementation, we developed short video tutorials. Fourth, since HCWs in high-burden settings sometimes lack access to internet or face drug stock-outs, these structural barriers should be addressed when introducing WGS into routine TB care.

This study also had several limitations. While our study is the first to solicit the perceptions and beliefs of HCWs with hands-on experience, their experience was limited to a pragmatic clinical trial. Future studies should explore the experiences when NGS is implemented in routine care. Our study was limited to a small number of HCWs in the Free State province in South Africa. Results may therefore not be generalizable to other contexts, particularly to health systems with different resource availability and diagnostic infrastructure. Furthermore, while the perspectives of policymakers, laboratory staff, and patients are also relevant and valuable, they have not been explored in this study. Finally, we did not use intercoder agreement metrics to assess analytic consistency, but attempted to supplement this by reaching consensus on codes and themes with the broader workgroup.

## Conclusion

Our study provides a nuanced real-world understanding of how *Mtb* NGS can be adopted in high-burden settings. The positive experiences expressed with the use of WGS when accompanied by automated treatment recommendations may pave the way for policymakers to explore the implementation of NGS in routine RR-TB care.

## Supporting information

S1 File Supplementary Material S1: Focus Group Discussion Guide. (DOCX)

## Abbreviations

BDQ: Bedaquiline
DST: Drug susceptibility testing
FQ: Fluoroquinolones
HCWs: Health care workers
LPA: Line probe assays
Mtb: Mycobacterium tuberculosis
NGS: Next-generation sequencing
RR-TB: Rifampicin-resistant tuberculosis
SMARTT: Sequencing Mycobacteria and Algorithm-determined Resistant Tuberculosis Treatment
WGS: Whole genome sequencing.
