# Supplementary material for: Potentials and challenges of next generation sequencing-guided individualized treatment for management of rifampicin-resistant tuberculosis – A qualitative study
Source: PLoS One. 2026 Jun 26;21(6):e0350514. doi: 10.1371/journal.pone.0350514 (PMC13308840; doi:10.1371/journal.pone.0350514)
Supplement: S1 File — (DOCX) [file pone.0350514.s001.docx]

**EVALUATION OF WGS -GUIDED TREATMENT RECOMMENDATION AND APP**

**Focus group with Physicians**

1. **Introduction**

*Thank you for your time and willingness to participate in the discussion. Has everyone signed the informed consent form?*

*My name is […]. We are social scientists at […]. We have been asked by the SMARTT team to discuss the SMARTT intervention with you. Today, in this focus group discussion, we will seek to understand your experience and opinions on the use of WGS, the automated treatment recommendation, and the app. We do this to better prepare the use of WGS in management of RR-TB patients outside of a research setting.*

*We will audio record the discussion for our analysis and then erase the recoding. The SMARTT trial team will not be able to link your name to your answers.*

*Do you have any question before we start?*

1. **Focus group guide**

*The use of WGS of sputum samples for patient with RR-TB is new. We would like to learn more about how you experienced the use of WGS in the context of the SMARTT trial*

**[general understanding, benefits, challenges]**

1. Can anyone give a description of WGS in their own words?
2. Can anyone give a description in their own words of how the automated treatment recommendation works?
3. Before we go into details, in general, has the combination of WGS, automated treatment recommendation and availability of the app delivered what you wanted or expected it to deliver?

**[benefits, challenges, risks]**

1. What do you consider to be the main benefits to doctors of WGS accompanied with an automated recommendation for RR-TB treatment regimen?

*Benefits as compared to the current strategy of 1^st^ and 2^nd^ line probe assay, pDST for selected drugs, and opinion of the provincial committee for complicated cases*

1. What do you consider are the main challenges for doctors to use WGS for RR-TB care?

*Challenges as compared to the current strategy of 1^st^ and 2^nd^ line probe assay, pDST for selected drugs, and opinion of the provincial committee for complicated cases*

1. In general, what do you think are the main risks when WGS and automated treatment recommendation is used for patient care

*Risk as compared to as compared to 1^st^ and 2^nd^ line probe assay, pDST for selected drugs, and opinion of the provincial committee for complicated cases*

*Does this affect the autonomy of a physician in decision making?*

*Could this reduce the competencies of physicians in managing RR-TB?*

**[Compatibility with current values and practice, trust]**

1. Compared to managing patients for whom WGS was not available, what was the impact of the availability of WGS and automated treatment recommendation?

*Workload: more or less time spent on treatment decision making*

*Difficulty and complexity of decision making*

*More or less communication with the provincial committee*

*Sense of responsibility*

1. To what extend do you trust the treatment recommendation?

*What is needed to enhance the trustworthiness?*

1. Have you ever doubted the treatment recommendation?

*If yes, can you describe the case(s) where you had doubt?*

1. Have you ever rejected the recommendation made by the treatment recommender?

*If yes, can you describe the case(s) where you rejected the recommendation?*

**[routine use of WGS and treatment recommender]**

1. Not taking costs into account, do you think WGS would be useful for all RR-TB patients or only for certain RR-TB patients?

*If for all patients: why?*

*If for select patients: why? which patients?*

1. Do you think that, when communicated with physicians, the WGS results should be accompanied by the automated treatment recommender?

*Why or why not?*

1. If the DOH, who want to limit costs, ask you for your choice between different strategies. Assume the price per patient for 1^st^ plus 2^nd^ line LPA is about 1200 Rand, the cost of WGS test about 1500 rand. Which strategy would you recommend?
   1. No LPA, WGS on early positive cultures for all RR-TB patients
   2. No LPA, WGS on early positive cultures for all RR-TB patients who have a history of TB treatment
   3. First do LPA, then WGS on early positive cultures if LPA shows resistance to RIF and INH, susceptible to FQ and injectables
   4. First do LPA, WGS on early positive cultures if LPA shows resistance to FQ

*Why?*

*Any other suggestions?*

**[Experience with the app – technological aspect, usefulness]**

*The research team also developed the treatment recommender* ***app****, so that doctors can, during a patient visit, consult the treatment recommender. We want to discuss your experiences with the treatment recommender* ***app****.*

1. For patient care, do you use any digital tools?

*If yes, which? what is your experience with those?*

1. What was your experience with the training for the treatment recommender app?

*How easy or difficult was it for you to learn how to use it?*

*Based on your experiences, what type of training is needed for doctors and nurses to use the treatment recommender?*

1. Have you ever faced difficulties when using the treatment recommender app?

*If yes, what was the difficulty you experienced and how was it solved?*

1. In your opinion, what are the risks of using the app?
2. In which scenario’s do you think it could be essential for doctors to have access to the app?

*Treatment failure, new stock out of drugs, development of drug toxicity*

1. To receive the treatment recommendation, which format do you prefer, the app or pdf form?

*Why?*

1. How could the pdf form be improved to be more useful the physicians?
2. How could the app be improved to be more useful for physicians?
3. What do you think would be the barriers for implementation of the app into routine care?

*Human factors, technology and infrastructural factors, organizational factors*

1. **Wrap up**

*Do you have any final remarks or questions?*

*Thank you so much for your time and your informative answers. Your participation has contributed a lot to our understanding of this subject.*

*I will end the recording now.*

*Thank you once again for your collaboration.*
