# Supplementary material for: Potentials and challenges of next generation sequencing-guided individualized treatment for management of rifampicin-resistant tuberculosis – A qualitative study
Source: PLoS One. 2026 Jun 26;21(6):e0350514. doi: 10.1371/journal.pone.0350514 (PMC13308840; doi:10.1371/journal.pone.0350514)
Supplement: Attachment — Submitted filename: Review comments.docx [file pone.0350514.s002.docx]

**Review comments**

**General comments**

This is a unique study which focused on next-generation-sequencing-guided individualized treatment for RR-TB and that has necessitated that a single focus group discussion study appear relevant. I would have wished that the Authors opted for a Key informant interview or in-depth interview approach bearing in mind the status of individuals that they included as participants. In any case, the study has its merits and I recommend a minor revision

Authors should bear in mind the following observations.

**Study title:** The title of the study is of two versions. The initial has two acronyms while the second has one acronym which is RR-TB and should be preferred.

**Participants’ profile:** The first paragraph in the result section should be sub-titled Participants’ profile.

**Themes:** The number of themes that emerged from the study was not stated and listed. After the sub-heading on participants’ profile, the next should be themes that emerged from the study.

**Journal guidelines**. Authors should ensure that they adhere to the guidelines of the Journal. For example, PLoS ONE uses square brackets for reference numbers instead of circular brackets. Authors should also ensure that the referencing style conforms to the guidelines of the Journal. Revision is required for reference number, 1, 4 and others.

**Conclusion:** Include conclusion as a sub-heading instead of using ‘In conclusion…’

**Reporting of results:** The abundance of quotes as presented by the Authors underscores the richness of the study findings. It is however expected that Authors should guide the readers properly with comments and remarks to support the flow of the quotes. In most instances, it is just quotes and quotes. The quotes should be presented in italics, with inverted commas and indented on both sides.

**Study approach:** It will be good for the Authors to explain why they preferred the FGD approach instead of embarking on a KII or IDI. Was it due to convenience or that they had the opportunity of having the study participants together at a particular point in time? An explanation is required. The duration of the FGD should be indicated.
