# Supplementary material for: Potentials and challenges of next generation sequencing-guided individualized treatment for management of rifampicin-resistant tuberculosis – A qualitative study
Source: PLoS One. 2026 Jun 26;21(6):e0350514. doi: 10.1371/journal.pone.0350514 (PMC13308840; doi:10.1371/journal.pone.0350514)
Supplement: Attachment — Submitted filename: FGD_SMARTT_Review comments_20260421.docx [file pone.0350514.s003.docx]

**Reviewer #1:** Major Comments

1. Clarify the sampling method (purposive, convenience, or census) and justify the adequacy of a single FGD

Answer: We thank the reviewer for the suggestion. In this study, total population purposive sampling was used to recruit all healthcare workers who had managed trial participants assigned to the WGS-guided and automated treatment recommendation RR-TB management. We used this sampling technique to ensure that FGD participants had direct experience with the phenomenon under investigation (WGS and the recommendation tool).

This approach was considered appropriate as participants shared similar roles and direct experience with the intervention, enabling collection of shared practices, consensus, as well as diverging perspectives on the phenomenon under investigation.

One single FGD was organized due to the small number (n=11) of eligible participants who agreed to participate in an in-person FGD.

We have added the above information to the Methods section (lines 68-75).

2. Provide additional details on FGD logistics: duration, mode (in-person/online), and key discussion topics.

Answer: We have added the following information to the Methods section (lines 77, 81-84) and Results section (line 112): The FGD was in-person and was conducted for one hour and 32 minutes. The discussion explored the individual, technological and organizational context and focused on experience with use of WGS-guided care and the treatment recommender, its perceived benefits and risk, and the facilitators and barriers of adopting the intervention in clinical practice.

3. The authors should explain how they handled differences in coding between the two analysts/researcher (TPHT and AB) and whether they checked or reported intercoder agreement to demonstrate reliability of the thematic analysis.

Answer: Two researchers (TPHT and AB) independently reviewed and coded the transcripts and subsequently discussed their interpretations to reconcile coding differences and agree on the final themes through consensus. We have revised the methods to better describe the process.

We appreciate the reviewer’s suggestion for the use of formal intercoder agreement statistics. We did not apply these statistics because the use of quantitative intercoder reliability measures in the analysis of qualitative research data is not yet standard in qualitative research. In response to this comment, we have carefully read on this topic (for example Nicholas Cofie et al. *Eight ways to get a grip on intercoder reliability using qualitative-based measures*, and Sean N. Halpin *Inter-Coder Agreement in Qualitative Coding: Considerations for its Use*) and appreciate the approach. We will surely implement these measures in our future qualitative analyses,. Unfortunately, individual preliminary coding records were not retained for this study. Retrospective assessment of intercoder reliability is thus not possible. We acknowledge this as a limitation of the analytic process and have added this clarification to the manuscript (Methods section line 89-90, Discussion section lines 299-300).

4. Add a short paragraph explaining who conducted and analyzed the FGD, their relationship to participants, and how they handled personal or professional biases to ensure a credible analysis.

Answer: We agree that researchers’ reflexivity is important in qualitative research. We have added that sub-section to the manuscript (lines 92-105), where we described the professional background, prior belief and potential biases of the interviewer and analysts, and how the credibility was ensured.

Minor Comments

5. Use consistent participant identifiers (e.g., P#1, Nurse/Physician) in quotations.

Answer: We have reviewed the manuscript to ensure that the participant identifiers are consistent. We prefer to refrain from including the job title in the participant identifiers because it may reveal participants’ identity due to the small number of participants.

6. Provide a short extract of the FGD guide or example questions as supplementary material.

Answer: We have now included the FGD guide as the supplementary material.

7. Expand slightly on limitations, focusing on transferability and contextual scope.

Answer: We thank the reviewer for this suggestion. We have now added to the Discussion section (lines 295-299) that because our studies concerned the experience of a group of health-care workers in a clinical trial setting in South Africa, the findings may have limited transferability to HCWs in other contexts, particularly those working outside trial setting or in health systems with different resource availability and diagnostic infrastructure.

**Reviewer #2:** The authors present a qualitative study on the perception of NGS-guided management of RR-TB among healthcare workers, based on a clinical decision support system tested in the SMARTT trial. I believe this is a valuable and timely study, as the field of diagnostics and management of TB, especially RR-TB, gradually moves towards more personalized treatments guided by genomics. The main limitation of the study is its qualitative nature and small sample size, which limits the capability of generalizing the observations beyond this particular setting. However, the authors properly acknowledge this limitation in the manuscript. I have no major concerns about the study, but I have some suggestions that I hope will help improving the manuscript.

Answer: We thank the reviewer for the positive remarks.

8. For the different testimonials, it is not clear to me how to interpret absence of comments, particularly because the full interview answers are not provided with the manuscript. For example, "according to the participants, the WGS simplifies the DST algorithm [...]". Then two testimonials (I#2) and (I#9) are provided. Would that mean that the 9 remaining HCWs did not think that WGS simplifies DST? Or they did not have any opinion on it?

Answer: Thank you for this observation. While inclusion of full transcripts as supplementary materials allow for full transparency we opted to not make these public because the small number of participants poses substantial risk to revealing the identity of FGD participants, even if anonymized. Rather than full transcripts, we have included the interview guide as supplementary materials and selected quotes within the main text. Due to the interactive nature of FGDs, not all participants comment on every topic. It is therefore not possible to have an exact quantification of how common a viewpoint was. The presented quotations are intended to illustrate analytically derived themes rather than to suggest that all participants expressed the same views and that a consensus was reached. Where opposing views emerged, these were explicitly described in the results. We have also put greater emphasis on making it clear to the readers that the statements are not necessarily consensus statement.

9. I believe that the authors have the chance of using an interesting approach in their qualitative research by using available LLMs, which I would normally not suggest, but seem to be appropriate for this kind of study. For example, the answers of the HCWs could be fed to an LLM (e.g GPT, DeepSeek, Gemini) to then summarize the experiences of the HCWs for the different sections. If appropriately documented in the methodology, including the models and prompts used, this would give the study a more objective perspective.

Answer: We agree that the LLMs is a promising tool for qualitative research. However, there is no consensus of whether LLM can be reliably trusted for qualitative data analysis. In fact, several papers have raised concern about the ethics and suitability of GenAI for qualitative research ^1,2^. GenAI could pose several epistemic risks to qualitative research, including the production of false and untrustworthy results, lack of contextual and tacit understanding, and the difficulty to evaluate the validity of its output. We therefore opted not to perform the analysis using LLMs.

10. In the discussion section it is stated that "The novel NGS-guided approach was viewed as evidence-based and dynamic, enabling HCWs to confidently switch from a standard regimen to an individualized treatment [...]". But I could argue that they do not seem so confident, citing (I#2) "Personally, there are a lot of those resistance patterns that I do not know. So, I am just given this information, and I have to trust what it says.", or (I#8), "I trust it to a certain extent, especially when the recommended treatment has what we call ‘powerful’ drugs in it, or drugs that are classified higher on the hierarchy of MDR drugs. And I trust it less when the so-called drugs which are lower on the classification", or even the own authors' words "HCWs were uncomfortable when the treatment recommendation deviated from the guidelines."

Answer: We thank the reviewer for this critical and constructive remark. We acknowledge that our initial wording in the discussion section did not fully reflect the content in the Results section. We have now adjusted the discussion to give a more balanced representation of the results (lines 245-248).

Minor comments:

10. "Currently, in the absence of rapid drug susceptibility tests (DST) for BDQ, a standardized treatment regimen is empirically started, and a rescue regimen is selected based on incomplete DST information when BDQ resistance is diagnosed." Are there references for this?

Answer: We have added the reference of the 2023 South Africa Guideline for clinical management of RR-TB^3^ to that statement. The guideline stated that Patients with resistance to LZD, CFZ and/or BDQ should start a rescue regimen that is recommended by the Provincial Clinical Advisory Committee, and extended DST is done on request by clinicians only to support individualized long regimen.

11. "Even when doctors have access to NGS, there is limited expertise in translating NGS results into individualized treatment regimens (8)." Reference 8 is 13 years old. Are doctors still as unfamiliar with NGS results as 13 years ago?

Answer: We thank the reviewer for the remark. We have added more recent references that highlighted the current challenges of translating NGS results to clinical decision. Particularly, Dookie et al. (2022) stated that the poor correlation between *Mtb* genomic and phenotypic drug susceptibility results is still hindering the interpretation of genomic data for clinical decision-making including selection of appropriate treatment regimen ^4^. There remains considerable uncertainty in the WHO catalogue of mutations for interpreting resistance data ^5^. Furthermore, the paper by de Araujo, L., et al. (2023) on implementing NGS for DR-TB diagnosis in Namibia also mentioned the need for a clinical advisory committee to provide guidance to clinicians based on reports generated by tNGS ^6^.

**Reviewer #3:** Review comments

**General comments**

This is a unique study which focused on next-generation-sequencing-guided individualized treatment for RR-TB and that has necessitated that a single focus group discussion study appear relevant. I would have wished that the Authors opted for a Key informant interview or in-depth interview approach bearing in mind the status of individuals that they included as participants. In any case, the study has its merits and I recommend a minor revision.

Answer: We thank the reviewer for the positive feedback.

Authors should bear in mind the following observations.

**12. Study title:** The title of the study is of two versions. The initial has two acronyms while the second has one acronym which is RR-TB and should be preferred.

Answer: we thank the reviewer for highlighting this and have deleted the acronyms from the title.

**13. Participants’ profile:** The first paragraph in the result section should be sub-titled Participants’ profile.

Answer: We have made the respective change.

**14. Themes:** The number of themes that emerged from the study was not stated and listed. After the sub-heading on participants’ profile, the next should be themes that emerged from the study.

Answer: We agree with the reviewer that there is value to the reader to first have an overview of the themes that emerged. In addition to the overview (lines 117-123), we have also added the numbering in the themes’ subheading.

**15. Journal guidelines**. Authors should ensure that they adhere to the guidelines of the Journal. For example, PLoS ONE uses square brackets for reference numbers instead of circular brackets. Authors should also ensure that the referencing style conforms to the guidelines of the Journal. Revision is required for reference number, 1, 4 and others.

Answer: We have revised the formatting of the manuscript to ensure that the guidelines are adhered to.

**16. Conclusion:** Include conclusion as a sub-heading instead of using ‘In conclusion…’

Answer: We have made the respective change.

**17. Reporting of results:** The abundance of quotes as presented by the Authors underscores the richness of the study findings. It is however expected that Authors should guide the readers properly with comments and remarks to support the flow of the quotes. In most instances, it is just quotes and quotes. The quotes should be presented in italics, with inverted commas and indented on both sides.

Answer: We thank the reviewer for the suggestion. We have revised the Results section, elaborated on the narration and interpretation to improve its flow.

**18. Study approach:** It will be good for the Authors to explain why they preferred the FGD approach instead of embarking on a KII or IDI. Was it due to convenience or that they had the opportunity of having the study participants together at a particular point in time? An explanation is required. The duration of the FGD should be indicated.

Answer: We thank the reviewer for this important question, and we agree that the rationale for performing the FGD should be stated. In our study, we considered FGD to be an appropriate option because of several reasons. First, this format allowed participants to build on one another’s comments, challenge differing perspective, and trigger deeper reflection. As a results, we could explore shared experiences, collective attitudes or disagreement among participants. Second, the discussed topics were not sensitive and the participants had similar professional roles, facilitating open discussion within a relatively homogeneous group. Third, considering the busy schedule of healthcare workers, FGD is more efficient than the other methods and we had a good opportunity to perform an FGD when participants gathered for a conference.

We have included the rationale in the Methods section (lines 69-75). The duration of the FGD is also mentioned on lines 112.

References:

1	Davison, R. M. *et al.* The ethics of using generative AI for qualitative data analysis. *Information Systems Journal* **34**, 1433-1439 (2024). <https://doi.org/https://doi.org/10.1111/isj.12504>

2	Nguyen, D. C. & Welch, C. Generative Artificial Intelligence in Qualitative Data Analysis: Analyzing—Or Just Chatting? *Organizational Research Methods* **29**, 3-39 (2026). <https://doi.org/10.1177/10944281251377154>

3	Department of Health - Republic of South Africa. (ed Republic of South Africa Department of Health) (2023).

4	Dookie, N., Khan, A., Padayatchi, N. & Naidoo, K. Application of Next Generation Sequencing for Diagnosis and Clinical Management of Drug-Resistant Tuberculosis: Updates on Recent Developments in the Field. *Front Microbiol* **13**, 775030 (2022). <https://doi.org/10.3389/fmicb.2022.775030>

5	World Health Organization. *Catalogue of mutations in Mycobacterium tuberculosis complex and their association with drug resistance, second edition*. Second edn, (2023).

6	de Araujo, L. *et al.* Implementation of targeted next-generation sequencing for the diagnosis of drug-resistant tuberculosis in low-resource settings: a programmatic model, challenges, and initial outcomes. *Front Public Health* **11**, 1204064 (2023). <https://doi.org/10.3389/fpubh.2023.1204064>
