# Supplementary material for: Potentials and challenges of next generation sequencing-guided individualized treatment for management of rifampicin-resistant tuberculosis – A qualitative study
Source: PLoS One. 2026 Jun 26;21(6):e0350514. doi: 10.1371/journal.pone.0350514 (PMC13308840; doi:10.1371/journal.pone.0350514)
Supplement: Peer review history

# Peer review: Decision Letter 0

**DOI:** 10.1371/journal.pone.0350514.r001

**Authors:** Igor Mokrousov

2 Dec 2025

PONE-D-25-40872

Potentials and challenges of next-generation-sequencing-guided individualized treatment for RR-TB management – a qualitative study

PLOS ONE

Dear Dr. Tu,

Thank you for submitting your manuscript to PLOS ONE. After careful consideration, we feel that it has merit but does not fully meet PLOS ONE’s publication criteria as it currently stands. Therefore, we invite you to submit a revised version of the manuscript that addresses the points raised during the review process.

==============================

Please revise as recommended by the reviewers, to improve clarity of description (e.g., sampling method and justify the adequacy of a single FGD) and interpretation. Adddress all comments.

==============================

Please submit your revised manuscript by Jan 16 2026 11:59PM. If you will need more time than this to complete your revisions, please reply to this message or contact the journal office at plosone@plos.org. When you're ready to submit your revision, log on to https://www.editorialmanager.com/pone/ and select the 'Submissions Needing Revision' folder to locate your manuscript file.

Please include the following items when submitting your revised manuscript:

- A rebuttal letter that responds to each point raised by the academic editor and reviewer(s). You should upload this letter as a separate file labeled 'Response to Reviewers'.
- A marked-up copy of your manuscript that highlights changes made to the original version. You should upload this as a separate file labeled 'Revised Manuscript with Track Changes'.
- An unmarked version of your revised paper without tracked changes. You should upload this as a separate file labeled 'Manuscript'.

If you would like to make changes to your financial disclosure, please include your updated statement in your cover letter. Guidelines for resubmitting your figure files are available below the reviewer comments at the end of this letter.

If applicable, we recommend that you deposit your laboratory protocols in protocols.io to enhance the reproducibility of your results. Protocols.io assigns your protocol its own identifier (DOI) so that it can be cited independently in the future. For instructions see: https://journals.plos.org/plosone/s/submission-guidelines#loc-laboratory-protocols. Additionally, PLOS ONE offers an option for publishing peer-reviewed Lab Protocol articles, which describe protocols hosted on protocols.io. Read more information on sharing protocols at https://plos.org/protocols?utm_medium=editorial-email&utm_source=authorletters&utm_campaign=protocols.

We look forward to receiving your revised manuscript.

Kind regards,

Igor Mokrousov, Ph.D., D.Sc.

Academic Editor

PLOS ONE

Journal requirements:

When submitting your revision, we need you to address these additional requirements.

1. Please ensure that your manuscript meets PLOS ONE's style requirements, including those for file naming. The PLOS ONE style templates can be found at

https://journals.plos.org/plosone/s/file?id=wjVg/PLOSOne_formatting_sample_main_body.pdf and

https://journals.plos.org/plosone/s/file?id=ba62/PLOSOne_formatting_sample_title_authors_affiliations.pdf

2. In the online submission form you indicate that your data is not available for proprietary reasons and have provided a contact point for accessing this data. Please note that your current contact point is a co-author on this manuscript. According to our Data Policy, the contact point must not be an author on the manuscript and must be an institutional contact, ideally not an individual. Please revise your data statement to a non-author institutional point of contact, such as a data access or ethics committee, and send this to us via return email. Please also include contact information for the third party organization, and please include the full citation of where the data can be found.

3. Please amend either the title on the online submission form (via Edit Submission) or the title in the manuscript so that they are identical.

If the reviewer comments include a recommendation to cite specific previously published works, please review and evaluate these publications to determine whether they are relevant and should be cited. There is no requirement to cite these works unless the editor has indicated otherwise.

[Note: HTML markup is below. Please do not edit.]

Reviewers' comments:

Reviewer's Responses to Questions

**Comments to the Author**

1. Is the manuscript technically sound, and do the data support the conclusions?

The manuscript must describe a technically sound piece of scientific research with data that supports the conclusions. Experiments must have been conducted rigorously, with appropriate controls, replication, and sample sizes. The conclusions must be drawn appropriately based on the data presented.

Reviewer #1: Yes

Reviewer #2: Partly

Reviewer #3: Yes

**********

2. Has the statistical analysis been performed appropriately and rigorously?

Reviewer #1: N/A

Reviewer #2: N/A

Reviewer #3: N/A

**********

3. Have the authors made all data underlying the findings in their manuscript fully available?

The PLOS Data policy requires authors to make all data underlying the findings described in their manuscript fully available without restriction, with rare exception (please refer to the Data Availability Statement in the manuscript PDF file). The data should be provided as part of the manuscript or its supporting information, or deposited to a public repository. For example, in addition to summary statistics, the data points behind means, medians and variance measures should be available. If there are restrictions on publicly sharing data—e.g. participant privacy or use of data from a third party—those must be specified.

Reviewer #1: Yes

Reviewer #2: No

Reviewer #3: No

**********

4. Is the manuscript presented in an intelligible fashion and written in standard English?

PLOS ONE does not copyedit accepted manuscripts, so the language in submitted articles must be clear, correct, and unambiguous. Any typographical or grammatical errors should be corrected at revision, so please note any specific errors here.

Reviewer #1: Yes

Reviewer #2: Yes

Reviewer #3: Yes

**********

5. Review Comments to the Author

Please use the space provided to explain your answers to the questions above. You may also include additional comments for the author, including concerns about dual publication, research ethics, or publication ethics. (Please upload your review as an attachment if it exceeds 20,000 characters)

Reviewer #1: Major Comments

1. Clarify the sampling method (purposive, convenience, or census) and justify the adequacy of a single FGD.

2. Provide additional details on FGD logistics: duration, mode (in-person/online), and key discussion topics.

3. The authors should explain how they handled differences in coding between the two analysts/researcher (TPHT and AB) and whether they checked or reported intercoder agreement to demonstrate reliability of the thematic analysis.

4. Add a short paragraph explaining who conducted and analyzed the FGD, their relationship to participants, and how they handled personal or professional biases to ensure a credible analysis.

Minor Comments

1. Use consistent participant identifiers (e.g., P#1, Nurse/Physician) in quotations.

2. Provide a short extract of the FGD guide or example questions as supplementary material.

3. Expand slightly on limitations, focusing on transferability and contextual scope.

Reviewer #2: The authors present a qualitative study on the perception of NGS-guided management of RR-TB among healthcare workers, based on a clinical decision support system tested in the SMARTT trial. I believe this is a valuable and timely study, as the field of diagnostics and management of TB, especially RR-TB, gradually moves towards more personalized treatments guided by genomics. The main limitation of the study is its qualitative nature and small sample size, which limits the capability of generalizing the observations beyond this particular setting. However, the authors properly acknowledge this limitation in the manuscript. I have no major concerns about the study, but I have some suggestions that I hope will help improving the manuscript.

For the different testimonials, it is not clear to me how to interpret absence of comments, particularly because the full interview answers are not provided with the manuscript. For example, "according to the participants, the WGS simplifies the DST algorithm [...]". Then two testimonials (I#2) and (I#9) are provided. Would that mean that the 9 remaining HCWs did not think that WGS simplifies DST? Or they did not have any opinion on it?

I believe that the authors have the chance of using an interesting approach in their qualitative research by using available LLMs, which I would normally not suggest, but seem to be appropriate for this kind of study. For example, the answers of the HCWs could be fed to an LLM (e.g GPT, DeepSeek, Gemini) to then summarize the experiences of the HCWs for the different sections. If appropriately documented in the methodology, including the models and prompts used, this would give the study a more objective perspective.

For example, in the discussion section it is stated that "The novel NGS-guided approach was viewed as evidence-based and dynamic, enabling HCWs to confidently switch from a standard regimen to an individualized treatment [...]". But I could argue that they do not seem so confident, citing (I#2) "Personally, there are a lot of those resistance patterns that I do not know. So, I am just given this information, and I have to trust what it says.", or (I#8), "I trust it to a certain extent, especially when the recommended treatment has what we call ‘powerful’ drugs in it, or drugs that are classified higher on the hierarchy of MDR drugs. And I trust it less when the so-called drugs which are lower on the classification", or even the own authors' words "HCWs were uncomfortable when the treatment recommendation deviated from the guidelines."

Minor comments:

"Currently, in the absence of rapid drug susceptibility tests (DST) for BDQ, a standardized treatment regimen is empirically started, and a rescue regimen is selected based on incomplete DST information when BDQ resistance is diagnosed." Are there references for this?

"Even when doctors have access to NGS, there is limited expertise in translating NGS results into individualized treatment regimens (8)." Reference 8 is 13 years old. Are doctors still as unfamiliar with NGS results as 13 years ago?

Sincerely,

Galo A. Goig

Reviewer #3: Review comments

General comments

This is a unique study which focused on next-generation-sequencing-guided individualized treatment for RR-TB and that has necessitated that a single focus group discussion study appear relevant. I would have wished that the Authors opted for a Key informant interview or in-depth interview approach bearing in mind the status of individuals that they included as participants. In any case, the study has its merits and I recommend a minor revision

Authors should bear in mind the following observations.

Study title: The title of the study is of two versions. The initial has two acronyms while the second has one acronym which is RR-TB and should be preferred.

Participants’ profile: The first paragraph in the result section should be sub-titled Participants’ profile.

Themes: The number of themes that emerged from the study was not stated and listed. After the sub-heading on participants’ profile, the next should be themes that emerged from the study.

Journal guidelines. Authors should ensure that they adhere to the guidelines of the Journal. For example, PLoS ONE uses square brackets for reference numbers instead of circular brackets. Authors should also ensure that the referencing style conforms to the guidelines of the Journal. Revision is required for reference number, 1, 4 and others.

Conclusion: Include conclusion as a sub-heading instead of using ‘In conclusion…’

Reporting of results: The abundance of quotes as presented by the Authors underscores the richness of the study findings. It is however expected that Authors should guide the readers properly with comments and remarks to support the flow of the quotes. In most instances, it is just quotes and quotes. The quotes should be presented in italics, with inverted commas and indented on both sides.

Study approach: It will be good for the Authors to explain why they preferred the FGD approach instead of embarking on a KII or IDI. Was it due to convenience or that they had the opportunity of having the study participants together at a particular point in time? An explanation is required. The duration of the FGD should be indicated.

**********

6. PLOS authors have the option to publish the peer review history of their article (what does this mean?). If published, this will include your full peer review and any attached files.

If you choose “no”, your identity will remain anonymous but your review may still be made public.

**Do you want your identity to be public for this peer review?**   For information about this choice, including consent withdrawal, please see our Privacy Policy.

Reviewer #1: No

Reviewer #2: **Yes:**   Galo A. Goig

Reviewer #3: **Yes:**   EDMUND NDUDI OSSAI

**********

[NOTE: If reviewer comments were submitted as an attachment file, they will be attached to this email and accessible via the submission site. Please log into your account, locate the manuscript record, and check for the action link "View Attachments". If this link does not appear, there are no attachment files.]

To ensure your figures meet our technical requirements, please review our figure guidelines: https://journals.plos.org/plosone/s/figures

You may also use PLOS’s free figure tool, NAAS, to help you prepare publication quality figures: https://journals.plos.org/plosone/s/figures#loc-tools-for-figure-preparation.

NAAS will assess whether your figures meet our technical requirements by comparing each figure against our figure specifications.

Attachment Submitted filename: Review comments.docx

# Author comment: Author response to Decision Letter 1

**DOI:** 10.1371/journal.pone.0350514.r002

21 Apr 2026

Reviewer #1: Major Comments

1. Clarify the sampling method (purposive, convenience, or census) and justify the adequacy of a single FGD

Answer: We thank the reviewer for the suggestion. In this study, total population purposive sampling was used to recruit all healthcare workers who had managed trial participants assigned to the WGS-guided and automated treatment recommendation RR-TB management. We used this sampling technique to ensure that FGD participants had direct experience with the phenomenon under investigation (WGS and the recommendation tool).

This approach was considered appropriate as participants shared similar roles and direct experience with the intervention, enabling collection of shared practices, consensus, as well as diverging perspectives on the phenomenon under investigation.

One single FGD was organized due to the small number (n=11) of eligible participants who agreed to participate in an in-person FGD.

We have added the above information to the Methods section (lines 68-75).

2. Provide additional details on FGD logistics: duration, mode (in-person/online), and key discussion topics.

Answer: We have added the following information to the Methods section (lines 77, 81-84) and Results section (line 112): The FGD was in-person and was conducted for one hour and 32 minutes. The discussion explored the individual, technological and organizational context and focused on experience with use of WGS-guided care and the treatment recommender, its perceived benefits and risk, and the facilitators and barriers of adopting the intervention in clinical practice.

3. The authors should explain how they handled differences in coding between the two analysts/researcher (TPHT and AB) and whether they checked or reported intercoder agreement to demonstrate reliability of the thematic analysis.

Answer: Two researchers (TPHT and AB) independently reviewed and coded the transcripts and subsequently discussed their interpretations to reconcile coding differences and agree on the final themes through consensus. We have revised the methods to better describe the process.

We appreciate the reviewer’s suggestion for the use of formal intercoder agreement statistics. We did not apply these statistics because the use of quantitative intercoder reliability measures in the analysis of qualitative research data is not yet standard in qualitative research. In response to this comment, we have carefully read on this topic (for example Nicholas Cofie et al. Eight ways to get a grip on intercoder reliability using qualitative-based measures, and Sean N. Halpin Inter-Coder Agreement in Qualitative Coding: Considerations for its Use) and appreciate the approach. We will surely implement these measures in our future qualitative analyses,. Unfortunately, individual preliminary coding records were not retained for this study. Retrospective assessment of intercoder reliability is thus not possible. We acknowledge this as a limitation of the analytic process and have added this clarification to the manuscript (Methods section line 89-90, Discussion section lines 299-300).

4. Add a short paragraph explaining who conducted and analyzed the FGD, their relationship to participants, and how they handled personal or professional biases to ensure a credible analysis.

Answer: We agree that researchers’ reflexivity is important in qualitative research. We have added that sub-section to the manuscript (lines 92-105), where we described the professional background, prior belief and potential biases of the interviewer and analysts, and how the credibility was ensured.

Minor Comments

5. Use consistent participant identifiers (e.g., P#1, Nurse/Physician) in quotations.

Answer: We have reviewed the manuscript to ensure that the participant identifiers are consistent. We prefer to refrain from including the job title in the participant identifiers because it may reveal participants’ identity due to the small number of participants.

6. Provide a short extract of the FGD guide or example questions as supplementary material.

Answer: We have now included the FGD guide as the supplementary material.

7. Expand slightly on limitations, focusing on transferability and contextual scope.

Answer: We thank the reviewer for this suggestion. We have now added to the Discussion section (lines 295-299) that because our studies concerned the experience of a group of health-care workers in a clinical trial setting in South Africa, the findings may have limited transferability to HCWs in other contexts, particularly those working outside trial setting or in health systems with different resource availability and diagnostic infrastructure.

Reviewer #2: The authors present a qualitative study on the perception of NGS-guided management of RR-TB among healthcare workers, based on a clinical decision support system tested in the SMARTT trial. I believe this is a valuable and timely study, as the field of diagnostics and management of TB, especially RR-TB, gradually moves towards more personalized treatments guided by genomics. The main limitation of the study is its qualitative nature and small sample size, which limits the capability of generalizing the observations beyond this particular setting. However, the authors properly acknowledge this limitation in the manuscript. I have no major concerns about the study, but I have some suggestions that I hope will help improving the manuscript.

Answer: We thank the reviewer for the positive remarks.

8. For the different testimonials, it is not clear to me how to interpret absence of comments, particularly because the full interview answers are not provided with the manuscript. For example, "according to the participants, the WGS simplifies the DST algorithm [...]". Then two testimonials (I#2) and (I#9) are provided. Would that mean that the 9 remaining HCWs did not think that WGS simplifies DST? Or they did not have any opinion on it?

Answer: Thank you for this observation. While inclusion of full transcripts as supplementary materials allow for full transparency we opted to not make these public because the small number of participants poses substantial risk to revealing the identity of FGD participants, even if anonymized. Rather than full transcripts, we have included the interview guide as supplementary materials and selected quotes within the main text. Due to the interactive nature of FGDs, not all participants comment on every topic. It is therefore not possible to have an exact quantification of how common a viewpoint was. The presented quotations are intended to illustrate analytically derived themes rather than to suggest that all participants expressed the same views and that a consensus was reached. Where opposing views emerged, these were explicitly described in the results. We have also put greater emphasis on making it clear to the readers that the statements are not necessarily consensus statement.

9. I believe that the authors have the chance of using an interesting approach in their qualitative research by using available LLMs, which I would normally not suggest, but seem to be appropriate for this kind of study. For example, the answers of the HCWs could be fed to an LLM (e.g GPT, DeepSeek, Gemini) to then summarize the experiences of the HCWs for the different sections. If appropriately documented in the methodology, including the models and prompts used, this would give the study a more objective perspective.

Answer: We agree that the LLMs is a promising tool for qualitative research. However, there is no consensus of whether LLM can be reliably trusted for qualitative data analysis. In fact, several papers have raised concern about the ethics and suitability of GenAI for qualitative research 1,2. GenAI could pose several epistemic risks to qualitative research, including the production of false and untrustworthy results, lack of contextual and tacit understanding, and the difficulty to evaluate the validity of its output. We therefore opted not to perform the analysis using LLMs.

10. In the discussion section it is stated that "The novel NGS-guided approach was viewed as evidence-based and dynamic, enabling HCWs to confidently switch from a standard regimen to an individualized treatment [...]". But I could argue that they do not seem so confident, citing (I#2) "Personally, there are a lot of those resistance patterns that I do not know. So, I am just given this information, and I have to trust what it says.", or (I#8), "I trust it to a certain extent, especially when the recommended treatment has what we call ‘powerful’ drugs in it, or drugs that are classified higher on the hierarchy of MDR drugs. And I trust it less when the so-called drugs which are lower on the classification", or even the own authors' words "HCWs were uncomfortable when the treatment recommendation deviated from the guidelines."

Answer: We thank the reviewer for this critical and constructive remark. We acknowledge that our initial wording in the discussion section did not fully reflect the content in the Results section. We have now adjusted the discussion to give a more balanced representation of the results (lines 245-248).

Minor comments:

10. "Currently, in the absence of rapid drug susceptibility tests (DST) for BDQ, a standardized treatment regimen is empirically started, and a rescue regimen is selected based on incomplete DST information when BDQ resistance is diagnosed." Are there references for this?

Answer: We have added the reference of the 2023 South Africa Guideline for clinical management of RR-TB3 to that statement. The guideline stated that Patients with resistance to LZD, CFZ and/or BDQ should start a rescue regimen that is recommended by the Provincial Clinical Advisory Committee, and extended DST is done on request by clinicians only to support individualized long regimen.

11. "Even when doctors have access to NGS, there is limited expertise in translating NGS results into individualized treatment regimens (8)." Reference 8 is 13 years old. Are doctors still as unfamiliar with NGS results as 13 years ago?

Answer: We thank the reviewer for the remark. We have added more recent references that highlighted the current challenges of translating NGS results to clinical decision. Particularly, Dookie et al. (2022) stated that the poor correlation between Mtb genomic and phenotypic drug susceptibility results is still hindering the interpretation of genomic data for clinical decision-making including selection of appropriate treatment regimen 4. There remains considerable uncertainty in the WHO catalogue of mutations for interpreting resistance data 5. Furthermore, the paper by de Araujo, L., et al. (2023) on implementing NGS for DR-TB diagnosis in Namibia also mentioned the need for a clinical advisory committee to provide guidance to clinicians based on reports generated by tNGS 6.

Reviewer #3: Review comments

General comments

This is a unique study which focused on next-generation-sequencing-guided individualized treatment for RR-TB and that has necessitated that a single focus group discussion study appear relevant. I would have wished that the Authors opted for a Key informant interview or in-depth interview approach bearing in mind the status of individuals that they included as participants. In any case, the study has its merits and I recommend a minor revision.

Answer: We thank the reviewer for the positive feedback.

Authors should bear in mind the following observations.

12. Study title: The title of the study is of two versions. The initial has two acronyms while the second has one acronym which is RR-TB and should be preferred.

Answer: we thank the reviewer for highlighting this and have deleted the acronyms from the title.

13. Participants’ profile: The first paragraph in the result section should be sub-titled Participants’ profile.

Answer: We have made the respective change.

14. Themes: The number of themes that emerged from the study was not stated and listed. After the sub-heading on participants’ profile, the next should be themes that emerged from the study.

Answer: We agree with the reviewer that there is value to the reader to first have an overview of the themes that emerged. In addition to the overview (lines 117-123), we have also added the numbering in the themes’ subheading.

15. Journal guidelines. Authors should ensure that they adhere to the guidelines of the Journal. For example, PLoS ONE uses square brackets for reference numbers instead of circular brackets. Authors should also ensure that the referencing style conforms to the guidelines of the Journal. Revision is required for reference number, 1, 4 and others.

Answer: We have revised the formatting of the manuscript to ensure that the guidelines are adhered to.

16. Conclusion: Include conclusion as a sub-heading instead of using ‘In conclusion…’

Answer: We have made the respective change.

17. Reporting of results: The abundance of quotes as presented by the Authors underscores the richness of the study findings. It is however expected that Authors should guide the readers properly with comments and remarks to support the flow of the quotes. In most instances, it is just quotes and quotes. The quotes should be presented in italics, with inverted commas and indented on both sides.

Answer: We thank the reviewer for the suggestion. We have revised the Results section, elaborated on the narration and interpretation to improve its flow.

18. Study approach: It will be good for the Authors to explain why they preferred the FGD approach instead of embarking on a KII or IDI. Was it due to convenience or that they had the opportunity of having the study participants together at a particular point in time? An explanation is required. The duration of the FGD should be indicated.

Answer: We thank the reviewer for this important question, and we agree that the rationale for performing the FGD should be stated. In our study, we considered FGD to be an appropriate option because of several reasons. First, this format allowed participants to build on one another’s comments, challenge differing perspective, and trigger deeper reflection. As a results, we could explore shared experiences, collective attitudes or disagreement among participants. Second, the discussed topics were not sensitive and the participants had similar professional roles, facilitating open discussion within a relatively homogeneous group. Third, considering the busy schedule of healthcare workers, FGD is more efficient than the other methods and we had a good opportunity to perform an FGD when participants gathered for a conference.

We have included the rationale in the Methods section (lines 69-75). The duration of the FGD is also mentioned on lines 112.

References:

1 Davison, R. M. et al. The ethics of using generative AI for qualitative data analysis. Information Systems Journal 34, 1433-1439 (2024). https://doi.org/https://doi.org/10.1111/isj.12504

2 Nguyen, D. C. & Welch, C. Generative Artificial Intelligence in Qualitative Data Analysis: Analyzing—Or Just Chatting? Organizational Research Methods 29, 3-39 (2026). https://doi.org/10.1177/10944281251377154

3 Department of Health - Republic of South Africa. (ed Republic of South Africa Department of Health) (2023).

4 Dookie, N., Khan, A., Padayatchi, N. & Naidoo, K. Application of Next Generation Sequencing for Diagnosis and Clinical Management of Drug-Resistant Tuberculosis: Updates on Recent Developments in the Field. Front Microbiol 13, 775030 (2022). https://doi.org/10.3389/fmicb.2022.775030

5 World Health Organization. Catalogue of mutations in Mycobacterium tuberculosis complex and their association with drug resistance, second edition. Second edn, (2023).

6 de Araujo, L. et al. Implementation of targeted next-generation sequencing for the diagnosis of drug-resistant tuberculosis in low-resource settings: a programmatic model, challenges, and initial outcomes. Front Public Health 11, 1204064 (2023). https://doi.org/10.3389/fpubh.2023.1204064

Attachment Submitted filename: FGD_SMARTT_Review comments_20260421.docx

# Peer review: Decision Letter 1

**DOI:** 10.1371/journal.pone.0350514.r003

**Authors:** Igor Mokrousov

15 May 2026

Potentials and challenges of next generation sequencing-guided individualized treatment for management of rifampicin-resistant tuberculosis – a qualitative study

PONE-D-25-40872R1

Dear Dr. Tu,

We’re pleased to inform you that your manuscript has been judged scientifically suitable for publication and will be formally accepted for publication once it meets all outstanding technical requirements.

Within one week, you’ll receive an e-mail detailing the required amendments. When these have been addressed, you’ll receive a formal acceptance letter and your manuscript will be scheduled for publication.

An invoice will be generated when your article is formally accepted. Please note, if your institution has a publishing partnership with PLOS and your article meets the relevant criteria, all or part of your publication costs will be covered. Please make sure your user information is up-to-date by logging into Editorial Manager at Editorial Manager® and clicking the ‘Update My Information' link at the top of the page. For questions related to billing, please contact billing support.

If your institution or institutions have a press office, please notify them about your upcoming paper to help maximize its impact. If they’ll be preparing press materials, please inform our press team as soon as possible -- no later than 48 hours after receiving the formal acceptance. Your manuscript will remain under strict press embargo until 2 pm Eastern Time on the date of publication. For more information, please contact onepress@plos.org.

Kind regards,

Igor Mokrousov, Ph.D., D.Sc.

Academic Editor

PLOS One

Additional Editor Comments (optional):

Reviewers' comments:

Reviewer's Responses to Questions

**Comments to the Author**

1. If the authors have adequately addressed your comments raised in a previous round of review and you feel that this manuscript is now acceptable for publication, you may indicate that here to bypass the “Comments to the Author” section, enter your conflict of interest statement in the “Confidential to Editor” section, and submit your "Accept" recommendation.

Reviewer #1: All comments have been addressed

Reviewer #2: (No Response)

**********

2. Is the manuscript technically sound, and do the data support the conclusions?

The manuscript must describe a technically sound piece of scientific research with data that supports the conclusions. Experiments must have been conducted rigorously, with appropriate controls, replication, and sample sizes. The conclusions must be drawn appropriately based on the data presented.

Reviewer #1: Yes

Reviewer #2: Partly

**********

3. Has the statistical analysis been performed appropriately and rigorously?

Reviewer #1: Yes

Reviewer #2: N/A

**********

4. Have the authors made all data underlying the findings in their manuscript fully available?

The PLOS Data policy requires authors to make all data underlying the findings described in their manuscript fully available without restriction, with rare exception (please refer to the Data Availability Statement in the manuscript PDF file). The data should be provided as part of the manuscript or its supporting information, or deposited to a public repository. For example, in addition to summary statistics, the data points behind means, medians and variance measures should be available. If there are restrictions on publicly sharing data—e.g. participant privacy or use of data from a third party—those must be specified.

Reviewer #1: No

Reviewer #2: No

**********

5. Is the manuscript presented in an intelligible fashion and written in standard English?

PLOS ONE does not copyedit accepted manuscripts, so the language in submitted articles must be clear, correct, and unambiguous. Any typographical or grammatical errors should be corrected at revision, so please note any specific errors here.

Reviewer #1: Yes

Reviewer #2: Yes

**********

6. Review Comments to the Author

Please use the space provided to explain your answers to the questions above. You may also include additional comments for the author, including concerns about dual publication, research ethics, or publication ethics. (Please upload your review as an attachment if it exceeds 20,000 characters)

Reviewer #1: Dear author, thank you for considering my previous comments. I have no further suggestions at this time.

Reviewer #2: The authors answered my questions and I do not have further comments. Regarding the use of LLMs, I only suggested it as an improvement for the manuscript. Although the explanation of why LLMs were not used was not really convincing, I acknowledge that it is not necessary for this study, which is qualitative and readers will read it and interpret it accordingly.

**********

7. PLOS authors have the option to publish the peer review history of their article (what does this mean?). If published, this will include your full peer review and any attached files.

If you choose “no”, your identity will remain anonymous but your review may still be made public.

**Do you want your identity to be public for this peer review?**   For information about this choice, including consent withdrawal, please see our Privacy Policy.

Reviewer #1: No

Reviewer #2: **Yes:**   Galo A. Goig

**********

# Editor report: Acceptance letter

**DOI:** 10.1371/journal.pone.0350514.r004

**Authors:** Igor Mokrousov

PONE-D-25-40872R1

PLOS One

Dear Dr. Trang,

I'm pleased to inform you that your manuscript has been deemed suitable for publication in PLOS One. Congratulations! Your manuscript is now being handed over to our production team.

At this stage, our production department will prepare your paper for publication. This includes ensuring the following:

* All references, tables, and figures are properly cited

* All relevant supporting information is included in the manuscript submission,

* There are no issues that prevent the paper from being properly typeset

You will receive further instructions from the production team, including instructions on how to review your proof when it is ready. Please keep in mind that we are working through a large volume of accepted articles, so please give us a few days to review your paper and let you know the next and final steps.

Lastly, if your institution or institutions have a press office, please let them know about your upcoming paper now to help maximize its impact. If they'll be preparing press materials, please inform our press team within the next 48 hours. Your manuscript will remain under strict press embargo until 2 pm Eastern Time on the date of publication. For more information, please contact onepress@plos.org.

You will receive an invoice from PLOS for your publication fee after your manuscript has reached the completed accept phase. If you receive an email requesting payment before acceptance or for any other service, this may be a phishing scheme. Learn how to identify phishing emails and protect your accounts at https://explore.plos.org/phishing.

If we can help with anything else, please email us at customercare@plos.org.

Thank you for submitting your work to PLOS ONE and supporting open access.

Kind regards,

PLOS ONE Editorial Office Staff

on behalf of

Dr Igor Mokrousov

Academic Editor

PLOS One
